# Microplastics and environmental effects: investigating the effects of microplastics on aquatic habitats and their impact on human health

**DOI:** 10.3389/fpubh.2024.1411389

**Published:** 2024-06-06

**Authors:** Aref Yarahmadi, SeyedeMozhgan Heidari, Parisa Sepahvand, Hamed Afkhami, Hadis Kheradjoo

**Affiliations:** ^1^Department of Biology, Khorramabad Branch, Islamic Azad University, Khorramabad, Iran; ^2^Pediatric Department, Mashhad University of Medical Sciences, Mashhad, Iran; ^3^Nervous System Stem Cells Research Center, Semnan University of Medical Sciences, Semnan, Iran; ^4^Cellular and Molecular Research Center, Qom University of Medical Sciences, Qom, Iran; ^5^Department of Medical Microbiology, Faculty of Medicine, Shahed University, Tehran, Iran; ^6^Laboratory Department, Buraimi Hospital, Buraimi, Oman

**Keywords:** microplastics, human health, sources, biodegradation, environment

## Abstract

Microplastics (MPs) are particles with a diameter of <5 mm. The disposal of plastic waste into the environment poses a significant and pressing issue concern globally. Growing worry has been expressed in recent years over the impact of MPs on both human health and the entire natural ecosystem. MPs impact the feeding and digestive capabilities of marine organisms, as well as hinder the development of plant roots and leaves. Numerous studies have shown that the majority of individuals consume substantial quantities of MPs either through their dietary intake or by inhaling them. MPs have been identified in various human biological samples, such as lungs, stool, placenta, sputum, breast milk, liver, and blood. MPs can cause various illnesses in humans, depending on how they enter the body. Healthy and sustainable ecosystems depend on the proper functioning of microbiota, however, MPs disrupt the balance of microbiota. Also, due to their high surface area compared to their volume and chemical characteristics, MPs act as pollutant absorbers in different environments. Multiple policies and initiatives exist at both the domestic and global levels to mitigate pollution caused by MPs. Various techniques are currently employed to remove MPs, such as biodegradation, filtration systems, incineration, landfill disposal, and recycling, among others. In this review, we will discuss the sources and types of MPs, the presence of MPs in different environments and food, the impact of MPs on human health and microbiota, mechanisms of pollutant adsorption on MPs, and the methods of removing MPs with algae and microbes.

## Introduction

**Figure 1:**
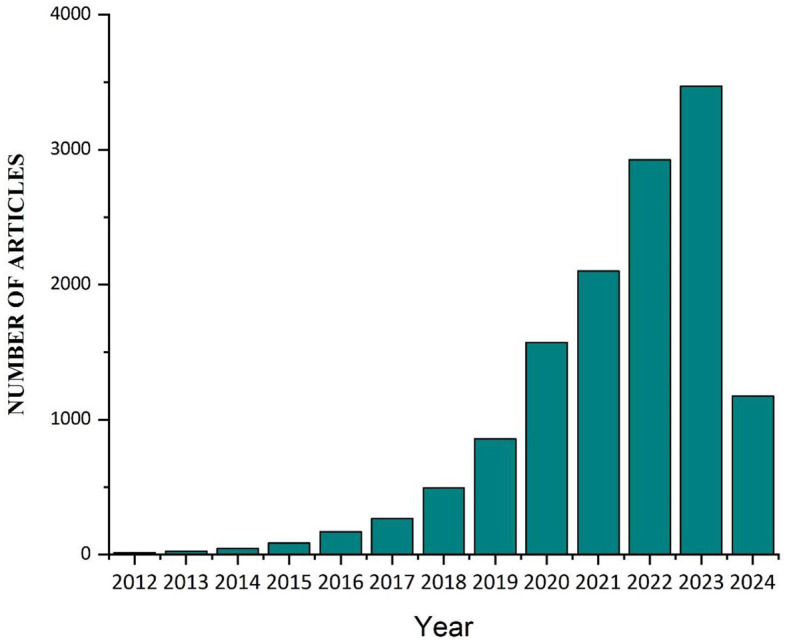
The number of articles published on the MPs updated to 22 March 2024. The expanding number of papers is indicative of the scientific community's and researchers' increased interest in and awareness of the problem of MP contamination. The number of papers has been steadily increasing, which highlights the relevance of tackling MP pollution in different ecosystems and the developing character of research on this topic.

There is growing concern among researchers and environmentalists regarding the impact of microplastics (MPs) on human health and aquatic ecosystems. They can be found in both freshwater and marine ecosystems, although the significance of freshwater environments is often overlooked and underreported in comparison to marine ecosystems (1, 2). MPs are particles with a diameter of <5 millimeters (3). The term “MPs” was introduced nearly two decades ago by Thompson et al. (4) in their research on marine plastic contamination in the United Kingdom. Since then, MPs have garnered the interest of the academic community, governmental bodies, non-governmental organizations, and various other stakeholders. In the last century, global plastic production has reached 320 million tons a year, and more than 40 percent of it is used as single-use packaging. One hundred ninety-two coastal nations produced 275 million metric tons (MT) of plastic garbage in 2010, and this figure reached 350 million tons in 2017 (5–8). In 2013, China manufactured around 63.0 million metric tons of plastic, representing the largest share of global plastic production. When this figure is aggregated with the plastic output of other nations in Asia, the collective plastic production amounts to around 114.0 million tons (9). The European Union emerged as the second most significant region in terms of plastic production, generating approximately 50.0 million tons (10). Furthermore, it is anticipated that the amount of plastic trash produced will reach around 250 million metric tons by 2025, placing further strain on systems for managing plastic garbage (11). The disposal of plastic waste into the environment poses a significant and pressing issue. Atmospheric elements, including abrasion, wave action, mild oxidation, and ultraviolet radiation, along with microbial activity, contribute to the degradation of plastic fragments into micro and nanoparticles (12, 13). There has been a notable rise in public apprehension regarding the issue of MP pollution in recent years. MPs can spread various unsettling deterrents through the environment in addition to acting as a poisonous deterrent. MPs impact the feeding and digestive capabilities of marine organisms, as well as hinder the development of plant roots and leaves (14–18). When plastics are introduced into the environment, they undergo degradation, resulting in the formation of smaller fragments that have the potential to enter the food chain directly or introduce potentially hazardous substances into it (19, 20). Most individuals ingest substantial quantities of MPs and even nanoplastic particles from their diet, particularly from consuming fish and other types of seafood (21). So far, MPs have been discovered in various food items, including drinking water, sugar, seafood, canned tuna, honey, and table salt (22, 23). Numerous nations have established goals to eradicate or decrease specific items like plastic bags and waste to combat plastic pollution (24, 25). In May 2018, the European Commission implemented a new plan that prohibited various single-use plastic items and imposed more stringent rules on others. Sixty nations have implemented prohibitions or levies on disposable plastics (26, 27). The generation and dispersion of plastic waste in marine environments are progressively escalating, leading to a corresponding increase in its accumulation at both surface and seabed levels (28). Plastic in aquatic environments disrupts vital conditions, leading to adverse impacts on the socio-economic aspects of industries such as tourism, trawling, shipping, and fish farming (29, 30). The resilient and enduring nature of MPs renders them prevalent in aquatic environments as a form of marine pollution, serving as a conduit for transmitting pollutants to aquatic organisms (31, 32). The diminutive dimensions of MPs result in their ingestion by various marine organisms, causing disturbances in their physiological processes. This subsequently permeates through the food chain, giving rise to adverse health effects in humans (33, 34). Exposure to MPs induces oxidative stress, microbial dysbiosis, and persistent inflammation within the human organism. These particles have also been associated with cancer and may also impact the development of the fetus (35–37). The number of articles published in PubMed with the Keyword microplastics [Title/Abstract] from 2012 to 22 March 2024 is shown in Figure 1.

The objective of this investigation is to acquire an extensive comprehension of MPs, encompassing their origin and assortment, along with exploring the significance of MPs in different environments and examining their influence on human health, as well as their impact on the microbiome and the degradation of MPs by algae, fungi, and bacteria.

## Types and sources of microplastics

**Figure 2:**
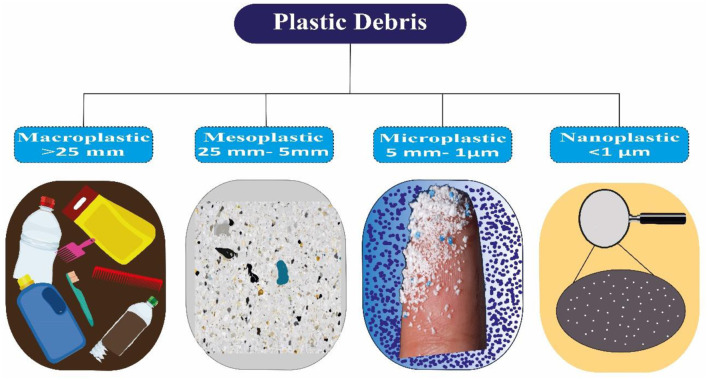
The size of all types of microplastics.

MP particles are categorized into many categories and vary in composition, size, color, and thickness. Plastic particles were classified as macro, meso, micro, and nano, respectively, based on their size ranges: more than 25 mm, between 5 and 25 mm, 1 to 5 mm, and 1 nm to 1 micrometer (38–40) (Figure 2). Research demonstrates two main sources of MPs: primary and secondary. Primary MPs are discharged into our ecosystems directly by tires, personal care items, marine coatings, and synthetic textile goods. Conversely, secondary MPs come from bigger plastics broken down into smaller pieces by mechanical abrasion or UV exposure. These larger plastics include fishing nets, garbage cans, and tire wear (41, 42). MP particles, with an average size of 0.25 mm, are commonly utilized in industrial shot-blasting abrasives and cosmetic treatments. Granules and powders, which are particles of MP diameters, are undoubtedly used in a variety of applications (43, 44). Primary MPs being released into the environment straight from home factories and sewerage. Skin care product MP beads are deposited in the environment due to being transported through the sewage system with sewage and not being adequately eliminated. An average of 700,000 fibers are released from 6 kg of synthetic garments containing MPs in a single wash (45–47). MPs enter the environment through pellets used as raw material for plastic products in industrial applications. Additionally,sewage releases MPs used in dentistry and pharmaceutical containers into the environment. Primary MPs are challenging to remove from aquatic systems because of their smaller size and lack of knowledge (28, 48). There has also been evidence of raw food contamination from contact with plastic chopping boards (49). The amount of MPs released from plastic materials may be influenced by several variables, including physical stress and heat. A polypropylene (PP) baby milk bottle heated from 25 to 90 degrees Celsius emitted between 0.6 and 55 million MP particles per liter (50). It is believed that MPs, which have also been recovered from surgical environments, are a result of the widespread usage of plastics in healthcare settings (51). There have also been reports of MPs falling from home objects. MPs have been found to have leaked from plastic tea filter bags, throwaway cups, and food containers (52–54). The prevalent MPs found in the environment include polyethylene (PE), PP, polyvinyl chloride (PVC), and polystyrene (PS) (55). Table 1 compiles the physical characteristics and origins of prevalent MPs (56).

**Table 1:** A set of physical characteristics and the origin of representatives of MPs (56–63).

**Plastic class**	**Size range**	**Density (g cm^−1^)**	**Items and common source**
High-density PE	0.06 to 11.06 μm	0.96	Solution containers, toys, tubing, gear
Low-density PE		0.92	Plastic wrap, bags, containers, medical products
PVC	Ranging from <10 nm to up to 20 μm	1.40	Pipe, containers, film, Medical equipment
PP	120 to 220 μm	0.90	Pipes, strapping, food packaging, microwavable lunch boxes, rope, drinking straws
PS	33 to 190 μm	1.02–1.05	floats, foamed foam, cups, insulation material, dish
Polyethylene terephthalate (PET)	12 to 18 μm in thickness Length: shorter than 1 mm	1.55	Fabric, water bottles, strapping, food packing
Polyamides (PA)	Between 20 and 50 μm	1.02–1.14	Fibers, fabric, carpet, pipe
Polyurethane (PU)	<5 mm	1.01–1.03	Adhesive, artificial leather, foam
Polycarbonate (PC)	5–200 nm	1.36	Medical tubes, instrument casings, insulators, eye lenses, dialysis equipment
Polymethylmethacrylate (PMMA)	———–	1.18	Eyeglass lens, Plexiglas, plates, orthopedics

## Microplastics in different environments

### MPs in marine environments

MP contamination has been documented in various ecosystems, including aquatic, terrestrial, and atmospheric environments (64–66). The passage describes the inevitable exposure of MPs in the human body due to the transfer of these particles through the aquatic food chain (67, 68). Most plastics have a density similar to that of water, causing plastic particles to either float or remain suspended in water and be carried by the current. According to a study, it was approximated that plastic constitutes 60 to 80 percent of marine debris (69). Elevated levels of plastic particles have been detected in coastal regions, fishing equipment, and semi-enclosed seas (70). Carson et al.' (71) research revealed that the levels of MP in the North Pacific gyre varied between 85 and 184 items per square kilometer. Lusher and colleagues (72) conducted a study in which they gathered and characterized 2,315 plastic particles from surface waters in the northeastern Atlantic Ocean. Their findings revealed that 89% of the collected particles were smaller than 5 mm, suggesting that MPs are the predominant form of plastic pollution in quantity (72). In the Yellow Sea of China, the mean concentration of MPs in the surface water was recorded at 545 ± 282 particles per cubic meter. Additionally, sea cucumbers, a commonly consumed food along China's coast, were found to ingest a higher quantity of MPs compared to other marine organisms in the same region (73). Meteorological events such as precipitation and severe weather can also influence the distribution and fate of MPs in the ocean to a certain degree (74). Hitchcock examined fluctuations in the prevalence of MPs in surface water in the context of a storm event and determined that the highest concentration of MPs reached 17,833 cubic meters at the height of the storm (75). The primary contributors to plastic fragments in marine ecosystems are activities that occur on land. The presence of MPs exhibited a notable positive association with population density and urban/suburban expansion. Additionally, MPs transported by coastal currents tend to amass near urban industrial hubs along the coastlines (76, 77). Furthermore, plastics are introduced into the marine ecosystem through direct means such as sewage overflows, sewage discharges, and shipping operations (78). MP contamination resulting from fishing activities is frequently underestimated. Large quantities of plastic materials are utilized in fishing equipment, including ropes, nets, and pots. Furthermore, the presence of fiber plastic waste can be attributed to the mechanical wear and tear of fishing nets and ropes during operational use (79). One research study found that the amount of plastic debris present on the surface of the ocean is significantly less than the total amount of plastic debris that enters the sea, as the majority of plastic waste ultimately settles on the ocean floor (80). After MPs are introduced into aquatic ecosystems, they do not solely remain as individual particles. Plastic particles that are suspended or floating in water engage with other substances or organisms through absorption, accumulation, and phagocytosis, leading to the vertical displacement of plastic particles within the water column (81). Furthermore, MP sedimentation is reversible, as even MPs located in deep water bodies or sediment can rise to the surface due to biological processes or water movement (82). The possibility that MP pollution might harm ecosystem health is one of the main worries (83). MPs' minuscule size makes them easy for marine animals to consume. When MP contaminants are eaten by smaller marine species (primary consumers, such as zooplanktons), then transferred to secondary consumers, such as big fish, and finally tertiary consumers, such as people, the food chain, or food web, is seriously disrupted (84). Meanwhile, when MPs reach the food chain, marine animals may end up eating additional pollutants and dangerous substances (such as poisonous heavy metal species) that stick to their surfaces or become lodged in their mass (85). The health of both people and marine life is at risk from these very toxic MPs. That is, the hydrophobic pollutants in seawater may stick to plastic debris in a typical environmental setting (86). Furthermore, MP pollution may have a significant impact on the biodiversity of the impacted ecosystems. MPs have the power to modify habitat structures, interfere with ecosystem functions, and affect an organism's ability to reproduce and behave (87, 88). For instance, MP buildup in aquatic environments can suffocate and smother benthic creatures, such as corals and shellfish, preventing them from growing and developing normally (89, 90).

### MPs in soil

Previous research has primarily concentrated on the dispersion of MPs in marine environments, but recent studies have underscored the significance of MPs in terrestrial ecosystems (91). MPs have been detected in soils across the globe, and the yearly influx of MPs into soil ecosystems may exceed that of the world's oceans (92). Agriculture represents a significant contributor to the presence of soil MPs, with an annual release of hundreds of thousands of tons of these particles into agricultural land (93). MPs have the potential to infiltrate agricultural soil via various pathways, such as the application of plastic mulch, compost, and irrigation. This can result in the buildup of numerous MPs, ranging from tens to hundreds per kilogram of soil (94, 95). Each year in Germany, an estimated 0.035 to 2.2 trillion MPs are introduced into agricultural soil through composting (96). The utilization of sludge resulted in the presence of 1,000–4,000 MP particles per kilogram of soil in farmland soil in Europe (97). Roughly 20 million hectares of agricultural land globally utilize plastic mulch, with China representing the majority share at approximately 90%. Removing the mulching film from agricultural fields requires significant labor and time, leading to instances where the films or their remnants are deliberately or inadvertently left behind in the farmland (94). MPs can potentially impact the biophysical characteristics of soil, including soil structure, pH levels, fertility, nutrient content, microbial activity, and the formation of water-stable aggregates (98). Currently, there is limited information regarding the potential response of plants to the presence of MPs. Recent research by de Souza Machado et al. (98) has confirmed that the addition of MPs changes the physical properties of soil, thereby affecting its hydrodynamics and microbial activity. This study also shows that the impact of MPs on the soil depends on the shape and size of the MP particles (94, 98). MPs build up in soils and affect terrestrial ecosystems, the soil biota, nutrient cycling, and soil biodiversity. When micro- and mesofauna in the soil are exposed to MPs, it can negatively impact their growth, reproduction, and overall ecosystem function. These pollutants can also be absorbed by plants, generating worries about human health consequences through the food chain (99, 100).

### MPs in freshwater

As previously stated, a significant portion of marine plastic originates from terrestrial sources, with rivers serving as the primary conduit for transporting plastics of diverse dimensions (101). The attention toward MPs in freshwater ecosystems is a relatively new development, with initial research published in the past 15 years. MPs have rapidly emerged as a prevalent form of pollution. It is not unusual for studies in freshwater environments to detect and document the presence of MPs at all sampling locations and frequently in all samples collected (102–104). The broadening of research attention to encompass freshwater ecosystems is paramount, given the recognized significance of rivers in the conveyance of MPs, especially toward marine habitats (105). MPs found in freshwater environments originate from diverse sources. Studies have indicated spatial associations between the types of MPs in a particular location and human activities. Significant contributors include industrial discharges, urban waterways, and effluents from wastewater treatment plants in the vicinity (55). Both direct and diffuse sources of pollution contribute substantial quantities of MPs to river ecosystems (106, 107). Scholars have approximated that the yearly release of MPs into adjacent rivers from an industrial manufacturing facility may reach a maximum of 95.5 tons (108). Since high tidal forces, among other mechanical consequences, worsen plastic disintegration in the marine environment, the freshwater environment is not susceptible to these forces, leading most experts to conclude that secondary MPs are primarily found in the latter. These are not all primary MPs, however, as examination of the morphology and particle size of MPs in freshwater environments has revealed that many are secondary MPs created by the breakdown of bigger plastics (109). Since secondary MPs can enter the freshwater ecosystem through various routes and can be formed either before or after entering freshwater, pinpointing a specific source can be challenging (110). An excellent illustration of secondary MPs introduced into the environment consists of synthetic fibers that become dislodged from clothing during the washing process. An average 6 kg wash load of acrylic fabric is estimated to release between 140,000 and 730,000 microfibers (47). The microfibers turn into secondary MPs before being released into the surroundings. However, most secondary MPs are created following their introduction into the environment via biological, photodegradation, or mechanical abrasion. They are then carried to the freshwater environment by wind, surface runoff, and other activities (105, 111).

### MPs in the atmosphere

Although polymers with higher densities have been recorded in atmospheric deposition/air mass sampling, the variety of polymer types detected in atmospheric samples described thus far does not clearly distinguish between lesser or greater density (112). Most MPs found in the atmosphere are microfibers with minor amounts of foam, film, and fragments. These MPs are mainly at the micron scale (113, 114). MPs found in urban air are frequently linked to high levels of human activity, with primary sources stemming from the incineration of waste, degradation of synthetic textiles, tire abrasion, industrial processes, and urban particulate matter (115, 116). The atmosphere is crucial in facilitating the movement of MPs. Additionally, the movement of air in the atmosphere and the processes of wet and dry deposition are significant mechanisms through which MPs originating from land-based sources may enter and impact other environmental compartments. These processes can influence the dynamics of plastic pollution as it moves between different ecosystems (112, 117). Furthermore, the distribution of atmospheric MPs is influenced by patterns of rainfall and heat cycles (118). This indicates that the movement and preservation of plastics in the air vary depending on the current weather patterns over various periods or geographical areas. The research above, has established the widespread presence of MPs in the atmosphere and the potential for terrestrial MPs to be carried into marine environments. However, the extent to which atmospheric transportation contributes to pollution in aquatic and terrestrial ecosystems remains uncertain. Additional investigation is necessary to explore the transportation mechanisms and their correlation with meteorological factors (119–121).

### MPs in human food items

The widespread presence of MPs in oceans and seas suggests that products derived from these marine environments may also contain significant amounts of MPs. Numerous studies have reported the occurrence of MPs in aquatic organisms, particularly in seafood such as crabs, fish, and clams (122, 123). Likewise, sea salt, a non-living product derived from the sea, has also been documented to contain MPs (124, 125). As per the research conducted by Danapoulos et al. (126), most studies have detected MP contamination in seafood, with reported MPs content typically being <1 MP particle per gram (126). In a study, Jin et al. (127) found that aquatic food items such as fish and bivalves exhibit varying levels of MPs, with concentrations ranging from 0 to 10.5 items per gram for bivalves and 0 to 20 items per individual for fish. These same authors reported that drinking water and salt are also a pathway of MPs exposure to humans, with concentrations ranging from 0–61 particles/L in tap water, from 0–3,074 MPs/L in bottled water, and from 0–13,629 particles/kg for salt (127, 128). MPs have also been detected in various food sources, including vegetables (6.4 particles/100 g), honey (1,992–9,752 particles/kg), sugar (249 ± 130 particles/kg), cereals (5.7 particles/100 g), fruits (5.2 particles/100 g), beers (152 ± 50.97 particles/L), dairy products (8.1 particles/100 g), meats (9.6 particles/100 g), energy drinks (14 ± 5.79 particles/L), tea (11 ± 5.26 particles/L), and soft drinks (40 ± 24.53 particles/L) (129–134). Sure researchers argue that the consumption of MPs through bottled water is typically higher than tap water (128, 135). A compilation of research results from multiple studies regarding the presence of MPs in seafood is presented in Table 2 (122, 136, 137, 139–143).

**Table 2:** MPs contamination in seafood.

**Fish species**	**Ecosystem**	**Country**	**MPs Shape**	**MPs type**	**Reference**
*Sciades sona*					
*Priacanthus hamrur*					
*Benthopelagic species*					
*Carangoides chrysophrys*					
*Otolithoides pama*	Marine	Bangladesh	Films, granules, foams, fragments, fibers	PP, PS, PE, PU, styrene-butadiene rubber (SBR)	(136)
*Harpadon nehereus*					
*Setipinna tenuifilis*					
*Anodontostoma chacunda*					
*Megalaspis cordyla*					
*Sardinella brachysoma*	Marine	Philippines	Foams, fragments, fibers, microbeads, pellets	———	(137)
*Kuhila rupestris*	Freshwater				
*Valamugil speigleri*					
*Mystus macropterus*					
*Cyprinus carpio*			Films, granules, flakes, fibers, foams, strings, lines	PVC, PP, PE, PET, PS, PA	(138)
*Pelteobagrus fulvidraco*	Freshwater	China			
*Pelteobagrus vachelli*					
*Cirrhinus molitorella*			Pellets, fragments, fibers	———	(139)
*Oreochromis niloticus*					
*Achirus mazatlanus*					
*Mugil curema*					
*Paralabrax maculatofasciatus*					
*Eucinostomus dowii*		Mexico	Fibers	———-	(140)
*Calamus brachysomus*					
*Balistes polylepis*	Marine				
*Sardinella albella*					
*Rastrelliger kanagurta*					
*Istiophorus platypterus*		India	Fibers	Polyester, PA, PE	(141)
*Harpodon nehereus*					
*Chirocentrus dorab*					
*Katsuwonus pelamis*					
*Commercially available fishes*			Pellets, fibers, films	PA, PE, PET	(142)
*Oyster/mussel*	Coastal environment	Korea	Fibers, fragments	Polyester, PA, PP, PE, PET, PS	(143)
*Manila clam*					
*Metapenaeus affinis*	Freshwater	Iran	fibers, films, fragments, spherules	PET, PS, PP	(144)

### The impact of MPs on human health

**Figure 3:**
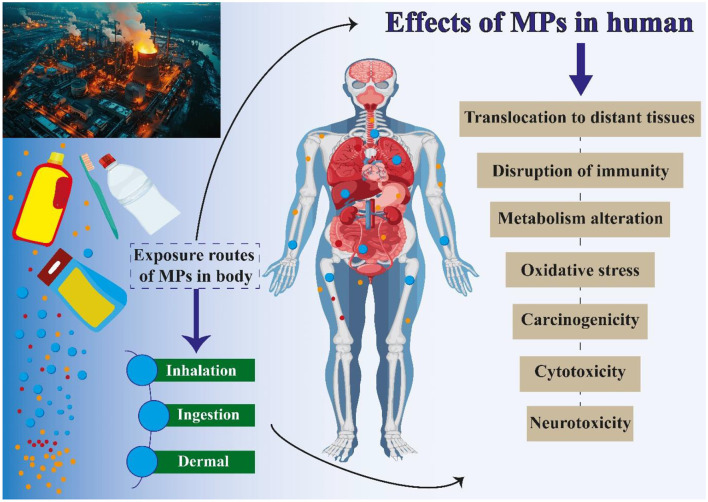
Routes of human exposure to MPs and the related risks.

As a burgeoning area of research, MPs necessitate further investigation to comprehensively understand their effects on both organisms and human health (145). Plastic production has significantly increased in recent years, with projections indicating an additional 33 billion tons of plastic will be generated by 2050, adding to the current level of approximately 370 million tons produced in 2019 (146). This remarkable surge in plastic production also serves as a cautionary reminder of the substantial volume of plastic waste being deposited into the environment (147). MPs have been discovered to possess the capacity to impact human health (148–150). Research has indicated that MPs have the potential to infiltrate the human body via ingestion of water and food, as well as inhalation of airborne particles (151). Also, MPs have been identified in various human biological samples, such as lungs, placenta, stool, sputum, liver, breast milk, and blood (148, 152). MPs can cause various illnesses in humans, depending on how they enter the body (Figure 3) (153). They may release harmful chemicals into the body, which may result in a variety of health concerns, including cancer, developmental disorders, and problems with reproduction (154, 155). Additionally, it has been discovered that MPs aid in developing antibiotic resistance. This is due to the possibility that MPs may operate as a breeding ground for bacteria that will eventually develop antibiotic resistance. Given that antibiotic resistance is already a major worldwide health problem, this might have substantial ramifications for human health (156, 157). Ingestion is the primary way that MPs are consumed. One of the main ways that people consume MPs is via eating seafood that has been polluted; eating sea salt can also cause one to consume MPs. The human body also obtains MPs from drinks, tap water, and bottled water. According to a recent study, MPs can enter the body by eating fruits and vegetables (158, 159). Inhaling dust from both indoor and outdoor sources also facilitates the entrance of MPs into the human body; the majority of MPs identified in dust come from synthetic fabrics, aerosols, and tires (160, 161). The direct penetration of MPs through the dermal layer is uncommon due to the delicate nature of the skin membrane. However, there have been documented instances of MPs entering through hair follicles, sweat glands, and skin lesions such as cuts or wounds (162). Recent research has indicated the presence of MPs in human fecal matter, providing evidence for the ingestion of MPs by humans (134, 163). The results of research on the effects of MPs on the human body are displayed in Table 3. The extent of penetration of MPs into the organs or lungs is influenced by the size of the MPs (135). The lungs or cells will directly absorb MPs that are a few microns in size through cellular uptake; more prominent MPs (up to 130 microns) can still reach tissues through paracellular uptake; MPs larger than 150 microns are not absorbed (135, 203). Hence, it can be inferred that MPs have the as well as to impact human health directly. In the following, we will examine the implications of MPs on human health, including the specific organs and tissues that may be affected.

**Table 3:** The consequences and functions of MPs on human organs.

**Affected organ/cell/tissue**	**Effects and illnesses**	**Reference**
Lungs and skin	Respiratory system lesions, decreased ventilatory capacity, skin disease, inflammatory reactions, dyspnea, genotoxicity, coughing, breathing problems, inflammation, bio persistence, respiratory systems, cancer, asthma, bronchitis, and cytotoxic effects.	(164–169)
Gastrointestinal tract	Colorectal Cancer, inflammatory bowel diseases, gut dysbiosis, Increased intestinal permeability, increased bacterial toxins, immune response, cytotoxicity, oxidative stress.	(170–179)
Ocular surface	Cytotoxic effects, reduced cell viability, inflammation, decreased tear volume, destruction of corneal epithelial microvilli, dry eyes	(180–183)
Reproductive and sexual system	Reduced sperm viability, disruption of endocrine processes and natural steroid hormones, oxidative stress, impaired gamete quality, developmental abnormalities, epigenetic changes, DNA damage	(184–195)
Cerebral cells	Nervous system issues, cytotoxic effects, neurotoxicity, abnormalities in brain development, inflammation	(196–199)
Placenta	Chemical absorption from plastic particle leaching	(200)
Cells and cell division	Respiratory system lesions, decreased ventilatory capacity, skin disease, inflammatory reactions, dyspnea, genotoxicity, coughing, breathing problems, inflammation, bio persistence, respiratory systems, cancer, asthma, bronchitis, and cytotoxic effects.	(201, 202)

### Lung damage

Previous research has identified the existence of synthetic fibers in human lung tissue samples. However, there is a scarcity of studies that have utilized chemical analysis techniques, such as micro-Raman spectroscopy and Fourier transform infrared spectroscopy, to definitively confirm the presence of MPs in the lungs (36, 204). The potential for inhaling MPs has been emphasized, with studies documenting the presence of MPs smaller than 5 μm in air samples (205). It is still uncertain whether MP particles can infiltrate and persist in the respiratory system of the general public due to environmental exposure, as opposed to the sustained levels observed in industrial environments. MPs are engineered to possess durability, making them resistant to lung degradation, which may result in their potential accumulation over time, contingent upon their aerodynamic diameter and the body's respiratory defenses (164, 206). Smaller-sized MPs can induce respiratory discomfort and cytotoxic and inflammatory effects when they penetrate the human respiratory system (164, 165, 207–209). Particles of 50 nm in size of PS have been shown to have cytotoxic and genotoxic effects on pulmonary epithelial cells (166). Interstitial fibrosis, inflammatory and fibrotic changes in the bronchial and peribronchial tissue (chronic bronchitis), and asthma-like bronchial reactions are a few symptoms of a synthetic particle entering the body. These symptoms have been seen in textile industry workers who are in close contact with acrylic, polyester, and nylon fibers (210). In comparison to bigger particles (202–535 nm), the smaller particles (64 nm) produced a considerably higher neutrophil influx in the lungs, according to research examining proinflammatory responses in rats to different sizes of PS particles (167). Evidence suggests that MPs may also spread to other tissues after being swallowed or breathed. For example, research reported that fluorescent PS microspheres given intraperitoneally to mice were discovered in the spleen 10 days later (211).

### Colorectal cancer

Colorectal cancer (CRC), which ranks as the third most prevalent cancer globally with 1.9 million cases reported in 2020, is on the rise among individuals under the age of 50 (212, 213). This rise is believed to be influenced by an environmental factor, and MPs have been investigated as a potential catalyst for this shift (170). CRC is associated with intestinal microbiota and their interaction with the mucosa (170). The current equilibrium between the gut bacteria and the mucus layer may be altered by MPs that enter the diet and make their way to the colon. Consequently, they may also modify the colonocytes' exposure to different potentially detrimental elements of the gut microbiota, thus impacting the occurrence of CRC. Moreover, colonocyte cells may be directly exposed to MP-associated carcinogens, raising the risk because MPs tend to adsorb hydrophobic compounds from the environment (170, 214). The discovery of significant MP concentrations in stool samples indicates that most MPs pass straight through the small intestine and into the colon. Newly discovered evidence of MPs in human blood and tissue samples indicates that not all MPs ingested are excreted directly through the gastrointestinal (GI) tract. Instead, considering that the dispersion of particles in the respiratory tract is largely dependent on particle size, it is probable that size may also impact the deposition, distribution, and, or aggregation of MPs at this location (171). Furthermore, surface-associated chemicals and, or porosity may affect how MPs are absorbed, distributed, and, or trafficked throughout the GI system. Because of the makeup of their polymers (PS, for example), most plastics have hydrophobic surfaces. MPs' chemical surface allows them to bind charged molecules and ions (including toxic metals) through electrostatic interactions, adsorb hydrophobic compounds (some of which are carcinogens), adhere microorganisms (some of which may be pathogens), and simply cause a local inflammatory response (which may lead to non-genotoxic carcinogenesis) (170). MPs eaten are likely to fragment due to a combination of factors, including GI fluids and mechanical pressures inside the GI lumen (215–220). Furthermore, the colon's chemical milieu, such as pH, may alter the properties of harmful chemical adsorption. This is probably especially important when it comes to ingesting MPs that have weathered. A few smaller MPs known as bile-associated MPs pass through the colon after being absorbed in the small intestine and ending in bile (221, 222). MPs will touch the loosely adhering outer mucous layer, which serves as the colon's initial line of defense as they transit through the body. This layer's hydrophobic domains can bind MPs, some of which may then be shed due to the GI tract's physiological peristaltic processes, which cause this layer to constantly turnover (223). The intestinal epithelium is probably similarly protected from MPs by the inner mucus layer, which typically serves as a barrier to shield the underlying epithelial cells from harmful substances and germs. When MPs penetrate the inner layer of the colonic mucus, they may serve as rafts for the growth of biofilms, which are intricate bacterial communities known to be significant regulators of gut health (170, 224). The hypothesis that bacterial adhesion to MPs *in situ* may offer a platform for early biofilm formation is supported by evidence of bacteria-rich biofilms on the surface of MPs recovered from seawater (225). Two recent investigations have examined MPs' potential for carcinogenicity, and both have concluded that there is probably a connection (172, 226). However, as Domenech et al. (226) note, most research has used short-term rodent trials or *in vitro* models, making it impossible to draw firm conclusions. Toxins produced by carcinogenic bacteria may be delivered to the colonic epithelium by MPs. This is demonstrated by developing a genotoxin by *Escherichia coli* (*E. coli*) in the colon, which is linked to an increased risk of CRC (173, 227). Although *E. Coli* typically lives in the intestinal lumen, research showing that *E. coli* can attach to MPs in an aquaculture model implies that they can also bind to MPs in the colon (228, 229). If this is the case, MPs containing pks+ *E. coli* have the potential to serve as a means of transporting these genotoxic bacteria to the colonic epithelium surface. However, evidence indicates that this process may rely on the absence of a fully intact inner mucus layer (230). Put simply, the relative abundance of pks+ *E. coli* next to the colonic epithelium may rise in response to a shift in the relative abundance of bacterial species (dysbiosis) that break down colonic mucus (231). Mice subjected to continuous exposure to MPs in their drinking water for 6 weeks also exhibited imbalanced microbial communities in the colon (174, 175). Given the growing body of evidence linking colonic microbiota to the development of CRC, this becomes increasingly significant (232, 233). Although the pro-carcinogenic mechanisms of these different bacterial species vary, it is plausible that their association with MPs could increase the delivery of the corresponding bacterial toxins to the colonic epithelium. This would support the theory that carcinogenesis in otherwise healthy colons may be caused by long-term storage of toxic bacteria (233, 234). Furthermore, research has demonstrated that MPs, specifically PE, can induce adverse effects in human intestinal cells, such as heightened oxidative stress and reduced cell viability (176).

### Inflammatory bowel disease

Following ingesting food and liquids, the gut, the most fundamental part of the human digestive system, carries out the crucial processes of digestion and nutrient absorption. Intestinal cells are exposed to various substances, poisons, and possible pollutants throughout this process. The intestinal barrier, which is made up of several components, has a variety of roles in immunological homeostasis and in preventing the entry of pathogens and toxins (235). Collectively, these elements work toward maintaining the proper operation of the digestive system and play a role in maintaining overall immune balance. As a result, the intestinal damage caused by contaminants poses a substantial threat to human wellbeing and is a matter of public apprehension. There is mounting evidence indicating that the disturbance or impairment of the intestinal barrier may play a role in the development of inflammatory bowel disease (IBD) and other systemic conditions (236–238). IBD is a non-specific, chronic GI illness that is typified by an immune response that is not normal. The two main types of IBD are Crohn's disease and ulcerative colitis (239, 240). The involvement of environmental variables in the development of IBD has been progressively validated by epidemiological research (241). A recent investigation revealed that the presence of MPs in the fecal matter of individuals with IBD was notably elevated at 41.8 items per gram of dry matter, in comparison to the lower concentration of 28.0 items per gram of dry matter in healthy subjects. Furthermore, a direct relationship was observed between the concentration of MPs and the severity of the disease (178). These findings imply that MPs have a significant role in the onset and course of IBD as an environmental factor. The fundamental processes behind the association between MP exposure and IBD are still unknown despite a lack of studies in this area (235). To ascertain the impact of MPs on the development of IBD, it is imperative to gain a comprehensive understanding of cytokines and their interplay with the pathogenesis of IBD (242). After cells are exposed to plastic particles, higher production levels of the cytokine interleukin 6 (IL-6) are seen. Furthermore, individuals with IBD exhibit elevated levels of IL-6 production by CD4+ T-cells and macrophages (167, 179, 243–246). The presence of MPs in the GI tract can disrupt the equilibrium of the intestinal immune system, leading to non-specific immune stress and impacting the integrity of the intestinal barrier (247). Liu et al. (248) conducted a study wherein they observed that 20 μg/mL polystyrene microplastics (PS-MPs) decreased the expression of TJ-related genes ZO-1 and Occludin and had a proinflammatory effect on colonic cell model Caco-2 cells. These findings suggest that PS-MPs can directly impact the structure and function of intestinal cells (248). Li et al. (243) discovered that feeding mice 600 μg/day PE-MPs for 5 weeks may raise the level of IL-1α in the serum and activate the signal pathways for TLR4/AP-1 (Toll-like receptor 4/Activated protein 1) and TLR4/IRF5 (Interferon Regulatory Factor 5) (243). Proinflammatory transcription factors that can stimulate inflammatory macrophage polarization, cell death, and cytokine production are activated by these pathways (249, 250). In the research conducted by Sun et al. (251), mice were given PE-MPs orally at doses of 0, 0.002, and 0.2 μg/g/d for 30 days. The investigators noted elevated levels of IL-1β and IL-6 in the high-dose group, suggesting that PE-MPs may induce a mild inflammatory reaction in the colon (251). Notwithstanding the absence of PS-MP accumulation in the gut, Rawle et al. (252) subjected mice to 80 μg/kg/d PS-MPs for 4 weeks and discovered substantial transcriptional alterations linked to inflammation in the colon (252).

### MPs and ocular surface

Research conducted *in vitro* demonstrated that human corneal and conjunctival epithelial cell lines could absorb MP particles made of PS, which would then gather around the nuclei of the cells. These particles were cytotoxic, as evidenced by the reduced cell viability and proliferation indicators (180). To investigate the effects of MP exposure on the ocular surface in mouse models, test animals were given 2.5 μL of a topical slurry containing 1 mg/mL of either 50 nm or two μm PS-MPs three times a day, without anesthesia, over 2–4 weeks. The control group was administered a normal saline treatment, while a separate group, referred to as the standard group, did not undergo any interventions (180). Weekly assessment of ocular surface fluorescein staining revealed a notable increase in staining within the test group, while no such increase was observed in the control or regular group. Interestingly, the mice receiving normal saline delivery showed intermittent punctate staining. The storage conditions of the normal saline, likely in a plastic container, were not specified, and there was no indication of pre-testing the normal saline solution for MPs. The production of tear film was examined weekly using a phenol red thread test. A decrease in the production of tears was observed, and this reduction in tear secretion persisted throughout the study in both treatment groups. Further evidence of a progressive build-up of MP particles in the lower conjunctival sac was provided by stereo-fluorescence microscopy. After the research, an analysis of *ex vivo* tissues revealed that, compared to the control group, the goblet cells in the lower lid had shrunk in size and density. In addition, there was a downregulation of proliferation-related markers (p63, Ki-67, and K14) in the treatment groups compared to the controls. In both treatment groups, the arrangement of lacrimal gland acini was different from that of the standard and control groups. There have also been reports of inflammatory cells between acini and time-dependent elevation of inflammatory factors and cytokines (IL-1α, IL1-β, and IL-6). Mice given the fluid containing 50 nm MP particles showed more excellent apoptosis rates than those given the suspension containing two μm MP particles (180). When particulate matter 2.5 (PM2.5) environmental pollutants, which may contain MPs, are exposed to the murine ocular surface, the result is decreased tear volume, a slower break-up of the tear film, and the loss of corneal epithelial microvilli and corneal desmosomes (181). Elevated concentrations of Tumor necrosis factor alpha (TNF-α) and Nuclear factor kappa B (NF-κB) p65 (Ser-536 phosphorylation) on the ocular surface indicated the presence of ocular surface abnormalities resembling those observed in individuals with dry eye disease (182). A recent multicenter cohort study conducted in China involving 387 individuals diagnosed with dry eye disease observed that areas with elevated levels of PM2.5 were associated with more severe Ocular Surface Disease Index (OSDI) scores, greater incidence of meibomian gland dysfunction, and elevated concentrations of IL-8 and IL-6 (183).

### MPs and male fertility

Numerous environmental pollutants have the potential to function as endocrine-disrupting chemicals (EDCs), imitating the actions of natural steroid hormones and disrupting endocrine processes through various mechanisms (184). Recently, there has been a notable focus on plastic additives, plasticizers, and emerging contaminants of concern (CECs), such as personal care products, pharmaceuticals, food additives, natural and synthetic hormones, and micro- and nano-sized. These substances are being released into the environment either directly or indirectly (184). In this regard, substances such as bisphenols, phthalates, poly- and perfluorinated alkyl substances, and others are widely utilized in the manufacturing of everyday consumer products, leading to their frequent release into the environment as waste (185). The adverse effects of EDCs include oxidative stress-induced tissue damage leading to apoptosis, developmental abnormalities, impaired gamete quality, metabolic disorders, neurotoxicity, and epigenetic changes due to *in-utero* exposure (185–189, 253). Research has indicated that exposure to MPs can lead to abnormalities in the structure of testicular and sperm cells, reduced sperm viability, and disruption of the endocrine system in male individuals (190). The harm inflicted by MPs on the male reproductive system may result in reproductive dysfunction and reduced fertility (191). The impact of the plasticizer bisphenol A (BPA) on spermatogenesis is multifaceted, involving central and local effects. It influences steroid biosynthesis, triggers apoptosis in germ and Sertoli cells, disrupts the initial stage of spermatogenesis, hinders the development of the blood-testis barrier, and alters the expression patterns of non-coding RNA, consequently impacting sperm quality (184). Various results regarding male reproductive effects have been documented about the method of exposure, levels of exposure, duration of exposure, and developmental stage. Research conducted on humans has compared levels of BPA in urine with semen parameters, suggesting a potential correlation between BPA exposure and decreased semen quality (192–194). For instance, Pollards et al. (193) demonstrated an increased exposure to BPA correlated with atypical sperm tail structure in a group of 161 men between the ages of 18 and 40 who did not have recognized subfertility. Omran et al. (194) documented an inverse relationship between urinary BPA concentrations and antioxidant levels, as well as semen quality parameters such as morphology, motility, and concentration. Additionally, they observed a positive correlation between BPA levels and DNA damage, as well as lipid peroxidation in seminal plasma. Finally, a potential association between the presence of BPA/phthalates metabolites in urine and sperm characteristics was examined, indicating a higher level of exposure to EDCs in individuals with reduced fertility compared to the broader population (195).

### Effects of MPs on microbiota

Healthy and sustainable ecosystems depend on the proper functioning of microbiota, with the diversity and quantity of microorganisms within a healthy microbiota believed to remain relatively constant (254). The microbiota comprises microorganisms that exhibit symbiotic, pathogenic, or commensal relationships. In multicellular organisms, the intestinal microbiota plays a crucial role in preventing diseases by creating a protective barrier against potential pathogens and enhancing GI physiology and mucosal immunity (255, 256). The utilization of mice as a model to investigate the effects of MPs on microbiota in organisms revealed that the absorption of PE led to an elevation in inflammatory markers such as IL-1β, IL-6, IL-8, and IL-10. Additionally, it resulted in a reduction in colon mucin expression, disturbance in lipopolysaccharide (LPS) metabolism, and an increase in the amino acid metabolism pathway of the microflora by modifying the composition of intestinal microflora (251). The microbiome plays a crucial role in preventing the introduction of novel bacterial strains from the surrounding environment through colonization. Disruption of this protective barrier could potentially facilitate the colonization of pathogens and contribute to the onset of disease (257). An instance of this is when Chinese mitten crabs (*Eriocheir sinensis*) were subjected to MPs, leading to the upregulation of immune-related genes and a reduction in the population of Firmicutes and Bacteroidetes, which are recognized as the predominant bacterial species in the GI tract (258). Members of Parliament MPs have been found to disrupt the balance of gut microbiota and induce inflammation in the intestines by promoting the growth of Proteobacteria and increasing LPS production in Danio rerio, a widely used aquatic model organism (259). Moreover, MPs have the potential to elevate the levels of reactive oxygen species (ROS) within various microorganisms, such as Danio rerio and Sparus aurata Linnaeus, by influencing the composition of bacterial populations in their microbiota, including Proteobacteria, Fusobacteria, Bacteroidetes, and Firmicutes (260, 261). Scholars have also documented the adverse effects of MPs on the growth and regeneration of epithelial cells in the intestinal tract of Danio rerio, a vertebrate species, through the reduction of *Pseudomonas* and *Aeromonas* populations (262). Within marine ecosystems, sediments serve as a primary reservoir of organic carbon, with the microbiota inhabiting these sediments playing a significant role in the biogeochemical processes and nutrient cycling within the ecosystem. Disturbing data indicates that MPs are disrupting the equilibrium of microbial communities in marine sediments. Seeley et al. (263) found that MPs have antibacterial properties that support certain types of bacteria, such as sulfate reducers, while hindering others like nitrifiers. Additionally, their research suggests that MPs could serve as a carbon source for specific microbial communities in sediment, such as *Acidobacteria, Bacteroidetes*, and *Chloroflexi* (264). Microorganisms functioning as decomposers play a crucial role in circulating organic compounds and energy within the soil ecosystem. However, introducing MPs into this ecosystem disrupts the equilibrium of bacterial populations. Research indicates that certain bacteria, such as *Rhodococcus ruber* and *Actinomadura* sp., can utilize MPs as a source of energy. However, the degradation process of MPs by these bacteria can lead to the release of harmful compounds like phthalates, which can adversely affect soil biota (260, 265). Moreover, due to the high hydrophobic nature of MPs, certain environmental pollutants like antibiotics and heavy metals tend to adhere to their surface through adsorption. The hazardous compound mixtures found in MPs have the potential to exert a more substantial influence on microbiota compared to the MP particles alone. A recent research study indicates that heavy metal concentrations found in MP particles are significantly higher, ranging from 10 to 100 times greater than those typically observed in the surrounding local environment (266). Furthermore, the microbiota balance is affected by the form, composition, and concentration of MPs. In this study, Sun et al. (91) examined the impacts of different concentrations and compositions of spherical MPs (150 μm) on the bacterial community within soil. The results indicated that the polymer structure's composition plays a significant role in influencing bacterial reactions within the soil environment (91).

### Mechanisms of pollutant adsorption on MPs

MPs serve as absorbers of pollutants in various environments because of their elevated surface area relative to their volume and their unique chemical characteristics (267, 268). When MPs break down into smaller plastic particles, more of their surface area is exposed, increasing their chemical reactivity, which might improve the adsorption of pollutants on MPs. Environmental factors that can have a substantial impact on the kinetics of contaminant adsorption onto MPs include weathering, UV, pH, and the hydrophobicity of persistent organic pollutants (POPs) (269). The efficiency of MP treatment and other emerging contaminants coexisting in the aquatic environment may be affected by MPs with adsorbed contaminants (c-MPs). However, studies on the mechanisms of contaminant adsorption on MPs, the fate and transport of MPs with adsorbed contaminants, and the effectiveness of MP treatment are often lacking. Per- and poly-fluoroalkyl substances (PFAS), one of the growing pollutants, have become a greater threat to human health due to their extensive use, manufacture, and resilience to environmental degradation (267, 270). Of all the perfluorinated compounds (PFCs), perfluorooctanoic acid (PFOA), and perfluorooctane sulfonate (PFOS) are particularly concerning due to their high stability, unclear destination, and frequent discovery in the environment, animals, and even human bodies (271). It's possible that newly identified pollutants of concern, such as per- and poly-fluoroalkyl substances (PFAS) polycyclic aromatic hydrocarbons (PAHs) and polychlorinated biphenyls (PCBs), will be adsorbed on MPs. Due to the widespread discovery of PFAS in drinking water, surface water, and wastewater treatment facilities, as well as their resistance to degradation and chemical stability, PFAS has recently been a significant source of worry (2, 272–281). PFAS consists of two primary categories, namely PFOA and PFOS, which have garnered escalating concern due to their adverse impacts on both public health and the environment (282). MPs readily absorb other POPs and pollutants (such as EDCs, PBDEs, and PPCPs) in aqueous conditions due to their hydrophobic nature. Because of their vast surface area and hydrophobicity, MPs may adsorb contaminants, which might lead to pollutants associated with MPs being released into the environment. The process of adsorption and desorption of pollutants onto and from MPs is intricate within diverse environmental settings due to a combination of dynamic variables including the characteristics of MPs (such as composition, structure, binding energy, and surface properties), the medium in which they are released (including pH, temperature, salinity, and ionic strength), and factors related to contamination (such as solubility, redox state, charges, and stability) (283–286). For instance, when benzo(a)pyrene is adsorbed onto PVC MPs, it exhibits a time- and dose-dependent adsorption kinetics that results in heightened toxicity levels compared to both unaltered MPs and benzo(a)pyrene in isolation. This underscores the substantial function of MPs as carriers for organic pollutants within sediment environments and highlights the potential synergistic impact of pollutant-absorbing MPs (287, 288). Under different environmental conditions, the pollutants could influence MPs' transition into byproducts including plastic particles, however, this information isn't documented in the literature. Regarding the processes of pollutants adsorbed on hydrophobic adsorbents, hydrophobic interaction, electrostatic repulsion and attraction, pore obstruction, and site competition may be the main mechanisms involved in c-MPs (289–292). MPs' hydrophobicity (K_OW_) and weathering/aging processes are the mechanisms by which contaminants adsorb onto them (293). Depending on the kind of MP, such as PE, PS, PP, and PVC, the adsorption/desorption kinetics may vary; PE (rubbery polymer PE) has greater adsorption than that of other forms of MPs (64). Even at high temperatures, a PFAS molecule with a negatively charged head and a hydrophobic C–F chain remains chemically stable (294, 295). The processes by which PFAS adhere to MPs may entail electrostatic and hydrophobic interactions, which play a significant role in the adsorption of PFAS onto various adsorbent substrates (296). Hydrogen bonding and covalent bonding can also be observed in the interactions between PFAS and adsorbents (296). The occurrence of either electrostatic repulsion or electrostatic interaction is dependent on the surface charge of adsorbents, with repulsion taking place when the adsorbent surface carries a negative charge, and interaction occurring when the surface charge is positive. For short-chain PFAS, electrostatic interactions seem to play a primary role, while longer PFAS tend to adsorb through hydrophobic interactions, promoting the formation of molecular aggregates of PFAS on the active surface of the adsorbent (297–299). The presence of organic matter (OM) in the environment can impact the adsorption of both long- and short-chain PFAS on MPs due to the complexation of PFAS with OM or co-sorption (296, 300). As a result, PFAS adsorption on OM in the presence of MPs may happen as a result of hydrophobic or electrostatic interactions between PFAS and OM-adsorbed MP surfaces (294, 296). The research found that OM inhibited the sorption of PFOA on active carbon fiber, but no discernible sorption happened when the quantity of OM was increased to 500 mg L^−1^ (301). The findings back up the competitive sorption between OM and PFOA as well as OM's pore-blocking of active carbon fiber. Through the clarification of the processes involved in the adsorption of pollutants onto MPs, a deeper comprehension can be gained regarding the destiny, movement, and environmental consequences of pollutants associated with MPs (267, 268, 292, 302).

### New methods of removing MPs

Numerous policies and initiatives exist at both the domestic and global levels to mitigate pollution caused by MPs. On a worldwide scale, the United Nations has initiated efforts to combat MP pollution by launching the Clean Seas campaign, which aims to eliminate primary sources of plastic and MP pollution in the oceans (303). The campaign is centered on advocating for the decrease and eradication of disposable plastics, enhancing waste disposal practices, and raising public consciousness (303, 304). In Europe, the European Union has enforced a prohibition on using MPs in personal care items, including facial cleansers and toothpaste (305, 306). The EU has also suggested implementing a prohibition on disposable plastics, encompassing items like utensils and drinking straws (307). Various strategies are used in MP removal and elimination procedures to address the problem of MP contamination (308). Studies on the degradation of MPs have advanced with a specific emphasis on biological and non-biological methodologies. The utilization of microorganisms such as algae, bacteria, and fungi for the degradation of MPs is viewed as a promising method for cost-efficient and environmentally friendly treatment strategies (309). The process of wastewater treatment is essential for the effective capture and removal of MPs from wastewater before its release into aquatic environments. Sophisticated treatment methods such as membrane filtration and activated sludge systems are utilized for this objective (308). Various filtration systems such as sand filters, mesh screens, and activated carbon filters are employed to capture larger MP particles from water sources. In regions characterized by elevated levels of MP accumulation, floating boom systems may be utilized to confine and retrieve floating plastic waste, which encompasses MPs. One commonly employed approach involves manually extracting visible plastic waste from rivers, shorelines, and beaches as part of clean-up efforts. Moreover, novel technologies such as electrocoagulation, magnetic nano adsorbents, and ultrasonic treatment are currently under investigation for their potential to improve the efficiency of removing MPs (310). The process of biological decomposition of MPs involves the presence of numerous enzymes (311, 312). Various extracellular enzymes such as lipases, esterases, laccases, lignin peroxidases, and manganese peroxidases are crucial in the degradation of MPs. These enzymes enhance the hydrophilicity of MPs and transform them into carbonyl or alcohol residues (313). Hydrolase enzymes, including esterases, lipases, and cutinases, facilitate the degradation of MPs on plastic surfaces by promoting chain cleavage reactions. These enzymes are unable to penetrate the polymer matrix. However, they exert their catalytic activity on the surface, leading to the development of fissures. The resulting monomers are absorbed into the cytoplasm of microorganisms and subsequently participate in various metabolic pathways (309). In the following, we will discuss novel methods for MP removal by algae, fungi, and bacteria.

### Algae in the degradation of MPs

Microalgae, along with their enzymes and toxins, have demonstrated efficacy in the enzymatic degradation of polymeric substances (314–316). One primary benefit is that they do not necessitate a high carbon source for their growth in contrast to bacterial systems, and they are well-suited to a diverse range of environments where most MPs are found (317). Microalgae have been observed to attach to plastic surfaces within wastewater streams, which leads to the initiation of plastic degradation through the secretion of ligninolytic and exopolysaccharide enzymes. Primarily, these polymers function as a carbon reservoir, augmenting cellular proteins, and carbohydrates, thereby enhancing the growth rate. Most recently, the surface deterioration or disintegration of low-density PE sheets due to algal colonization has been detected through scanning electron microscopy (SEM) (318). Algal biodegradation primarily occurs through, including hydrolysis, corrosion, fouling, penetration, and other processes (315). *Phormidium lucidum* and *Oscillatoria subbrevis* were identified as capable of inhabiting the surface of low-density polyethylene and breaking it down without the need for prooxidative additives or prior treatment (319). The compound BPA, which exhibits estrogenic properties and is frequently present in polymers, was decomposed through a collaborative effort involving various bacteria and algae species such as *Chlorella fusca var*. *vacuolate, Stephanodiscus hantzschii, Chlorella vulgaris*, and *Chlamydomonas mexicana* (320–322). Recent advancements in various biotechnological methods have enabled the development of genetically modified microalgal cell factories that can produce and release enzymes necessary for the degradation of plastics (323). The green microalgae *Chlamydomonas reinhardtii* underwent genetic modification to express Polyethylene terephthalate (PET) hydrolase, an enzyme capable of breaking down PET films and terephthalic acid (324). A comparable alteration was effectively implemented in *P. tricornutum*, resulting in the production of PET hydrolase that exhibited catalytic efficacy toward PET and the copolymer polyethylene terephthalate glycol (PETG) (314). In conclusion, microalgae may use plastic monomers as a carbon source by producing degrading enzymes, and because they are simple to grow, they have the potential to be effective MP degraders (325).

### Fungal degradation of MPs

The fungi encompass various organisms that primarily function as saprotrophs, opportunistic parasites, or obligate parasites. They exhibit remarkable adaptability and are capable of thriving in a variety of habitats, including aquatic and terrestrial ecosystems, across a range of environmental conditions. In addition to their ability to withstand harmful chemicals and metals, these organisms exhibit a wide array of external enzymes and natural surfactants, such as hydrophobins, which can break down intricate polymers into basic monomers. This process enables them to serve as a supplier of electrons and carbon for microorganisms, thereby aiding in the breakdown and conversion of complex pollutants into simpler forms (326, 327). The primary genera linked to the decomposition of various polymer varieties like PE, PET, and PP consist of *Cladosporium, Aspergillus niger, Zalerion maritimum*, and *Penicillium simplicissimum* (328–330). These microorganisms utilize MPs as their exclusive carbon source after the breakdown enable by extracellular enzymes. They facilitate the creation of various chemical bonds characterized by carboxyl, carbonyl, and ester functional groups while reducing their hydrophobic nature. The deterioration of PU material was observed in multiple fungal species, including *Cladosporium pseudocladosporioides, Aspergillus tubingensis, Aspergillus fumigatus, Penicillium chrysogenum*, and *Fusarium solani*, and in strains of *Pestalotiopsis microspora* (331–334). In most instances, serine hydrolase serves as a crucial factor in the process of PU degradation. The breakdown of high-density PE in marine coastal environments by two fungal strains, *Aspergillus tubingensis* VRKPT1 and *Aspergillus flavus* VRKPT2, was found to be approximately 6.02 ± 0.2% and 8.51 ± 0.1%, respectively (330). In a recent study, Kunlere et al. (335) documented the effective breakdown of low-density PE by *Aspergillus flavus* and *Mucor circinelloides* strains obtained from a municipal landfill. Before biodegradation, the MPs, specifically PE, can be subjected to pretreatment using substances like sodium hydroxide and nitric acid. This process has been observed to enhance the biodegradation rate of PE by the fungus *Aspergillus niger* (336). Thermal oxidation at 80°C for 15 days was necessary to induce degradation in low-density PE facilitated by *Penicillium pinophilum* and *Aspergillus niger*, resulting in degradation levels of 0.57% and 0.37% respectively following a 30-month incubation period (337). Likewise, *Lysinibacillus* spp. and *Aspergillus* spp. exhibited a biodegradation rate of 29.5% for UV-irradiated polymer films and 15.8% for non-UV-irradiated polymer films (338).

### Bacterial degradation of MPs

Various research investigations have been carried out utilizing bacteria to break down MPs. Bacteria with the ability to break down MPs have been identified in multiple environments, such as sludge, wastewater, polluted sediments, compost, municipal landfills, and even in extreme climates like Antarctic soils, mangrove areas, and marine sediments (339, 340). Additionally, microorganisms capable of degrading MPs have been identified within the GI microbiota of earthworms. It's commonly known that microorganisms that reside in contaminated areas frequently learn how to activate the enzyme system that breaks down MPs (341). Both individual bacterial strains and mixed bacterial communities can be employed to degrade MPs. Nevertheless, using pure cultures provides numerous benefits in the degradation process, serving as a practical method for investigating the metabolic pathways associated with this process. Furthermore, the influence of environmental elements such as pH, temperature, substrate properties, and surfactants on the degradation process can be more readily observed (342). The initial investigation into MP biodegradation by microorganisms was carried out by Cacciari et al. (343), who utilized a combination of *Pseudomonas stutzeri, Pseudomonas chlororaphis*, and *Vibrio* sp. to facilitate the degradation of PP. Similarly, the study also found that the inclusion of starch was observed to enhance the biodegradability capacity. Subsequent studies by Arkatkar et al. (344), as well as Fontanella et al. (345), documented the biodegradation of PP through the utilization of a mixed culture comprising *Pseudomonas stutzeri, Rhodococcus rhodochrous, Bacillus subtilis*, and *B. flexus*. In a research investigation by Auta et al. (346) *B. gottheilii* caused weight reductions of 6.2%, 3.0%, 3.6%, and 5.8% for PE, PET, PP, and PS MPs, respectively (347). Several other bacteria linked to the degradation of PP were identified, such as *Pseudomonas, Bacillus, Chelatococcus*, and *Lysinibacillus fusiformis*. These bacteria were isolated from diverse environments, including mangrove habitats, cow dung, compost, and land polluted with plastic waste. The intestinal microbiota of various arthropods such as *Plodia interpunctella* (Indian meal moth), *Tenebrio molitor* (mealworms), and *Galleria mellonella* (wax moths) have been documented to contain microorganisms with the ability to biodegrade MPs (348–350). In a research investigation by Yang et al. (348), a bacterial strain known as *Exiguobacterium* sp. was extracted from the intestinal tracts of mealworms, demonstrating the capacity to create biofilm structures and break down PS material. Effective degradation of low-density PE was achieved through the utilization of bacterial strains such as *Pseudomonas aeruginosa* and *Microbacterium paraoxydans*, resulting in degradation rates of approximately 61.0% and 50.5%, respectively, over 2 months under incubation conditions (351). Likewise, it has been documented that the biofilm produced by *Pseudomonas* sp. AKS2 can break down low-density PE by approximately 5 ± 1% over a 45-day incubation period without the need for any prior treatment (352). Similarly, the breakdown of PE was documented through the isolation of *Rhodococcus ruber* C208, with a degradation rate of 0.86% per week (353). The microorganisms obtained from the GI tract consisted of *Firmicutes* and *Actinobacteria genera*. These microorganisms were individually investigated and found to possess the capability to break down low-density PE MPs, leading to the release of volatile compounds such as docosane, eicosane, and tricosane. A collaboration between *Pseudomonas* and *Enterobacter* bacteria found in cow dung resulted in a weight reduction of up to 15% over 120 days (354). Numerous marine hydrocarbon-degrading bacteria, including *Alcanivorax borkumensis*, have demonstrated proficiency in breaking down alkanes, alkyl cycloalkanes, isoprenoid hydrocarbons, and branched aliphatic compounds (355). The study was conducted using the identical strain that had previously demonstrated the ability to form biofilms on low-density PE when exposed to hexadecane, pyruvate, and yeast extract, as well as on low-density PE films (356). Various actinomycetes, such as *Streptomyces* and *Rhodococcus ruber*, were also found to play a role in the biodegradation of PE (357). In the context of MP degradation, it was observed that *Pseudomonas* accounted for 21% of the bacterial genera involved, while *Bacillus* constituted approximately 15%. Additionally, combining these two genera contributed to 17% of the total bacterial population associated with this process (358).

## Conclusion

MPs are any type of plastic piece with a length of <5 millimeters, which has become one of the main challenges for the environment and public health. Studies have shown that MPs are found in various ecosystem environments, including marine, air, soil, and freshwater environments, and may enter the food chain. The influences of MPs on oceanic life and other ecosystems are significant, including ingestion by marine animals, interference with their reproductive systems, and even death. The harmful effects of MPs on human health are also severe. Studies have shown that MPs can be influential in various diseases and health complications, including damage to the lungs, eyes, brain, GI system, skin, male fertility, etc. They could discharge dangerous substances into the body, which might lead to several health issues, such as cancer, developmental difficulties, and reproduction issues. In addition, healthy and sustainable ecosystems depend on the proper functioning of microbiota; MPs cause damage to microbiota and upset their balance, which is a severe issue. However, new methods have been proposed to remove MPs, which include the use of algae, fungi, and bacteria. These approaches offer the potential to remove MPs from various environments and can be regarded as viable strategies for addressing this issue. A comprehensive comprehension of the source, classifications, impacts, and remedies associated with MPs is imperative to develop enhanced approaches for mitigating their adverse consequences on the environment and human health, thereby facilitating positive outcomes in this domain. The creation and implementation of strict environmental regulations aimed at managing and reducing MP contamination need to be a top priority for legislators. A few examples of these policies would be to outlaw single-use plastics in specific industries, provide incentives for recycling and reusing recyclable materials, enact laws to reduce the number of MPs released into the environment and promote the widespread use of biodegradable plastics. Scientists and researchers contribute significantly to our growing knowledge of the origins, distribution, and effects of MPs. Research projects examining non-plastic alternatives, creating cutting-edge technologies for MP identification and removal, carrying out long-term studies evaluating the health and environmental impacts of MP pollution, and lessening the harm that MPs cause to the ecosystem should all receive funding and support.

## Author contributions

AY: Investigation, Software, Writing – original draft. SH: Writing – original draft. PS: Writing – original draft. HA: Conceptualization, Project administration, Supervision, Writing – review & editing. HK: Conceptualization, Project administration, Supervision, Writing – review & editing.
